# Validation of the Malay Version of Mini-IPIP among Substance Use Disorder Patients Attending Methadone Clinics in Malaysia

**DOI:** 10.3390/ijerph16224434

**Published:** 2019-11-12

**Authors:** Foo Weng Leong, Mohd Azhar Mohd Yasin, Eni Rahaiza Muhd Ramli, Nor Asyikin Fadzil, Yee Cheng Kueh

**Affiliations:** 1 Department of Psychiatry, School of Medical Sciences, Universiti Sains Malaysia, Kubang Kerian 16150, Malaysia; lamblfw@gmail.com (F.W.L.); mdazhar@usm.my (M.A.M.Y.); drasyikin@usm.my (N.A.F.); 2 Hospital Universiti Sains Malaysia, Universiti Sains Malaysia, Kubang Kerian 16150, Malaysia; 3 Department of Psychiatry and Mental Health, Hospital Taiping, Taiping 34000, Malaysia; enirahaiza@gmail.com; 4 Unit of Biostatistics and Research Methodology, School of Medical Sciences, Universiti Sains Malaysia, Kubang Kerian 16150, Malaysia

**Keywords:** validity, reliability, structural analysis, short personality questionnaire, confirmatory factor analysis

## Abstract

There has been an increasing interest in personality study over the years. This has led to the necessity for personality measures with good psychometric properties. However, good personality measures are usually too cumbersome to apply in real practical settings due to their length. This study aims to validate a commonly used short personality measure of the Big Five model, i.e., Mini-IPIP (Mini International Personality Item Pool), which has never been validated and used in the substance abuse population in the local setting. The participants were 239 individuals attending one of the six methadone clinics in Malaysia. Structural analysis was conducted using confirmatory factor analysis. Results showed a good model fit for Mini-IPIP when item-parcelling and adding-in correlated uniqueness items were applied (fit indices: Comparative Fit Index = 0.949, Standardised Root Mean Residual = 0.044). Our study supported the five-factor solution for the Mini-IPIP. It is valid and reliable to be used among individuals with drug abuse in Malaysia.

## 1. Introduction

According to the diagnostic and statistical manual of mental disorders (DSM-5), personality refers to the “enduring patterns of perceiving, relating to and thinking about the environment and oneself that are exhibited in a wide range of social and personal contexts” [1]. Every person differs in their personality which makes them unique in their own way. The interest for personality study has grown over the years as evidenced by the number of personality-related empirical studies published over time. This is no surprise due to the increasing number of discoveries made regarding personality and its influences on human behaviour. Areas which are pertinent and closely related to the subject of personality are general and mental health, education, sports, work performance and many more. 

The Five Factor Model (also known as the Big Five Model) has been accepted as the dominant model to study personality in trait psychology [2,3]. This model incorporates the five personality traits or dimensions (i.e., intellect/openness, conscientiousness, extraversion, agreeableness and neuroticism) that are said to contain the facets or the building blocks that make up each trait or dimension. Several measures have been developed with the purpose to study the personality and individual differences among the people. The most established and well-studied personality measure with reported excellent psychometric properties (i.e., validity and reliability) is the Revised NEO Personality Inventory (NEO-PI-R) by Costa and McCrae [4], which has 240 items covering six facets for each of the five personality dimensions it assesses. Even so, it may be at a disadvantage in terms of its practical application as it takes approximately 45 minutes to complete the assessment. Respondents may get tired, bored and frustrated at having to complete the long questionnaire [2,5] which may lead to their random and inconsistent responding [6] and hence the questionable data quality [5]. Therefore, many researchers attempted to create a shorter version of personality measure hoping to come up with a concise form of the measure yet retain the excellent psychometric properties of their longer counterparts. Although shorter questionnaires are more practical in many situations such as during large-scale surveys or repeated-measure experiments, researchers using them have to compromise on their weaker psychometric properties [5,7] as shorter forms are associated with weaker validity and reliability. However, these shorter questionnaires can be used to assess the Big Five personality factors [7].

One of the short Five Factor Model instruments that has been established for many years and was used in the present study is the Mini International Personality Item Pool (Mini-IPIP). Mini-IPIP was developed by Donellan et al. [2] with the intention to create a short form of the 50-item IPIP established by Goldberg (1999). Through their five successive studies, a 20-item questionnaire with four items per factor, the questionnaire was formed and they showed good content coverage, test-retest correlations, validity and reliability. Other efforts to test its psychometric properties subsequently or to validate it to be used in local settings have shown mixed results [7]. For example, Cooper et al. [8] tested the psychometric properties of Mini-IPIP using confirmatory factor analysis (CFA) and found that the 5-factor model had “poor to moderate” model fit (CFI = 0.82, RMSEA = 0.07, SRMR = 0.06). Laverdière, Morin et al. [9] found that the initial CFA for the 5-factor model Mini-IPIP was suboptimal (CFI = 0.890, TLI = 0.870, RMSEA = 0.088). The model was subsequently modified by adding the items with correlated uniqueness which resulted in improved and satisfactory model fit (CFI = 0.944, TLI = 0.932, RMSEA = 0.064). 

The use of Big Five personality measures including the Mini-IPIP has been extensive on a variety of contexts including substance abuse [7,10,11] which is also the focus of this study. Direct relationships between certain personality traits and substance abuse have been proven in many studies [12,13]. For example, those with lower conscientiousness and agreeableness, and higher neuroticism, extraversion and openness are associated with substance use disorder [14]. Personality traits are also found to differ among substance abusers who use different types of illicit drugs. Terracciano et al. [15] noticed that individuals abusing cocaine and heroin scored very high in neuroticism and very low in conscientiousness as opposed to marijuana abusers who scored high on openness, average on neuroticism and low on agreeableness and conscientiousness. It was also found in a study that personality disorder manifestations, especially Cluster B types, in adolescence were associated with substance use disorder later in adulthood [16]. 

From the treatment point of view, Fisher et al. [17] reported that conscientiousness and neuroticism are two personality traits that are important to determine the rate of substance relapse. They found that those with low conscientiousness and high neuroticism have highest risk of relapsing. Therefore, the knowledge of personality dimensions of individuals involved in illicit substance use is relevant in the formulation of individualised treatment plan that matches each personality profile in which it has become an increasingly concerned area [15]. 

Personality study in Malaysia is still in its infancy. There are only a limited number of studies in the subject of personality in the area of substance abuse and even fewer studies on psychometric measures on personality in general. Therefore, the objective of the present study was to translate and validate Mini-IPIP among substance use disorder population in the local setting. The validation of this questionnaire would provide an impetus for more future research on personality measures with their associated topics especially in the area of substance abuse thus expanding the knowledge on the ever complex human behaviour. 

## 2. Materials and Methods

### 2.1. Study Design, Recruitment, and Sampling

**Figure 1:**
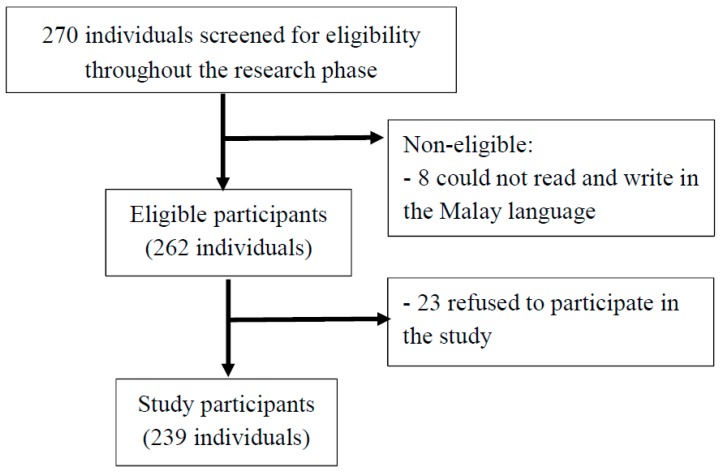
Participant recruitment into the study.

A cross-sectional study was employed, and the participants were recruited in the study after obtaining their consent. The study samples were collected from six methadone clinics in Malaysia. The participants were sampled using the convenience sampling method and were recruited into the study if they fulfilled the eligibility criteria of the study. Those included in the study were at least 18 years old and able to read and write in the Malay language. Those with concurrent active psychiatric illness or deaf and/or blind were excluded from the study. Flow chart of participant recruitment is illustrated in Figure 1. 

### 2.2. Participants

A total of 239 participants were involved in the study. The descriptive statistics for the sociodemographic particulars are shown in Table 1. The majority of the participants were male (95.8%) and Malay (91.6%). More than half of them were full-time working adults (61.5%) and had completed secondary level of education (85.4%). Also, the majority of the participants (48.1%) were still single at the time of study. Their age range was from 19 to 63 years with the male mean age of 39.0 years (SD = 0.61) and the female mean age of 35.8 years (SD = 2.70). 

### 2.3. Procedures

This study had gained approval from the Universiti Sains Malaysia Human Research Ethics Committee. This study also had conformed to the guidelines set by the International Declaration of Helsinki. Approval from the National Institute of Health Malaysia was also acquired at the same time. Upon volunteering, the participants were briefed about the objectives and procedures of the study. A set of personality questionnaires in the Malay language was given to the participants which included the Mini-IPIP and Zuckerman–Kuhlman Personality Questionnaire (ZKPQ). Participants then completed the questionnaires given and returned to the researcher (first author) on the same day of their study participation. Questionnaires were checked for their completion solely by the first author and hence any form of discrepancy during the sample collection was reduced to a minimum.

### 2.4. Materials

a. Mini-IPIP

Mini-IPIP by Donellan et al. [2] has 20 items with five subscales, i.e., Intellect, Conscientiousness, Extraversion, Agreeableness and Neuroticism, and each item is measured using a 5-point Likert scale from 1 (Very inaccurate) to 5 (Very Accurate). Each subscale is represented by four questions and they are divided equally into forward and reverse scorings except the intellect dimension where one question is for forward scoring while the other three are for reverse scoring. The Cronbach alphas for each of the personality dimensions are 0.82 (Extraversion), 0.77 (Agreeableness), 0.74 (Conscientiousness), 0.78 (Neuroticism), 0.70 (Intellect/Imagination). As for its validation using CFA, its CFI was 0.88 and the RMSEA was 0.07 (*p* close fit < 0.05) [2]. 

b. ZKPQ

Zuckerman–Kuhlman Personality Questionnaire Cross-Cultural 50 Items (ZKPQ-50-CC) by Aluja et al. [18] was an adaptation of the longer parental measure, i.e., Zuckerman–Kuhlman Personality Questionnaire (ZKPQ) by Zuckerman (2002). The original ZKPQ-50-CC’s CFA fit indices were CFI = 0.78, RMSEA = 0.04, and SMSR = 0.01 and the Cronbach alpha values for the English version were 0.80 (Neuroticism-Anxiety), 0.72 (Impulsive-Sensation-seeking), 0.74 (Activity), 0.74 (Sociability), and 0.72 (Aggression-Hostility). ZKPQ-50-CC was translated into Malay language and validated locally by Mohammad, Nadiah, and Geshina [19]. In their study, ten items (two from each factor) were removed and the remaining 40 questions in the five factors had Cronbach alpha coefficient values ranging from 0.76 to 0.84. Just like the original version of ZKPQ-50-CC, the translated version, i.e., ZKPQ-M-40-CC, has five common factors. Each factor has four items and each item is measured using a 5-point Likert scale. In this study, ZKPQ-M-40-CC was used during concurrent validation of Mini-IPIP. To our knowledge, this ZKPQ is the shortest validated Malay personality questionnaire to date.

### 2.5. Translation of Mini-IPIP

Mini-IPIP translation was done following the recommended steps from the WHO webpage under the research tools section [20]. It first underwent forward translation by an independent mental health professional and a layman. The two translated version questionnaires produced were back-translated by another independent mental health professional and another independent layman to assess the accuracy of the forward translations done earlier. The two forward Malay language translations were merged to produce the first consensus Malay version of Mini-IPIP after revision was done. Then, two mental health experts were involved in its content and face validity in which each item in the questionnaire was examined to ensure its suitability to be used in the Malaysian context. The harmonised version was produced after appropriate amendments were made. It was then used in the pre-testing stage of the study. Necessary adjustments were done to the questionnaire to produce the final version of the translated questionnaire. 

### 2.6. Data Analysis

The CFA in this study was conducted using the Mplus version 7.4 software program [21]. The statistical indices that we used to indicate the model fit were the comparative fit index (CFI), the Tucker–Lewis Index (TLI), the root mean square error of approximation (RMSEA), and the standardized root mean square residual (SRMR). The value of ≥ 0.95 is required for CFI and TLI to indicate good model fit whilst for RMSEA and SRMR, the acceptable value is ≤ 0.08 [22,23]. While we acknowledged that a priori 5-factor model would show the best model fit as proven in many studies, we also tested three other models to determine which one had the best model fit. Besides the 5-factor model, we had tested the 2- and the 3-factor models and the final model in which we performed the aggregate scores for each factor. 

As Cooper et al. [8] had tested on their 2- and 3-factor models, we similarly adopted the same factor structures for both our models. The 2-factor structure was based on the factors extracted by Digman [24] in which neuroticism, agreeableness and conscientiousness are loaded into alpha-factor while extraversion and intellect are loaded into beta-factor. Based on the values of the model fit indices obtained, we then removed items with poor factor loading (< 0.30) in stages and then applied the strategy of adding items with correlated uniqueness. This strategy has been practiced by a few authors, for example Laverdière et al. [9] and Marsh et al. [25] and the former was able to improve their CFA model fit of the Mini-IPIP after the strategy was applied. 

For the 3-factor structure, neuroticism and extraversion were grouped into one factor, conscientiousness and agreeableness into another factor, leaving intellect as a stand-alone factor. We then used similar steps and strategies like those for 2-factor model. Note that we also removed items with poor factor loading in stages and using the addition of items with correlated uniqueness in our 5-factor model. In our final model, we aggregated the scores for the items in each factor and then added in the items with correlated uniqueness. No item was removed in the final model and all the items remained as they were in their respective factor. We also perform reliability analysis for all items in the Mini-IPIP. 

We tested the concurrent validity of Mini-IPIP using the ZKPQ as the gold standard. Pearson correlation was used to examine the correlation between the factors within Mini-IPIP and ZKPQ.

## 3. Results

### 3.1. Structural Analysis for Mini-IPIP

Confirmatory factor analysis was performed in which the initial 5-factor model showed very poor model fit (see Table 2; CFI/TLI = 0.013/−0.137, RMSEA = 0.141, SRMR = 0.232). Items with poor factor loading (< 0.30) were then removed in stages with subsequent addition of items with correlated uniqueness (i.e., #15 and #10, #18 and #8, and #12 and #2). However, the final 5-factor model still showed poor model fit (CFI/TLI = 0.600/0.472, RMSEA = 0.102, SRMR = 0.132). For the 3-factor model, the initial model showed poor model fit (CFI/TLI = 0.358/0.283, RMSEA = 0.112, SRMR = 0.253). Items with poor loading were removed and items with correlated uniqueness were added in. The final model fit for 3-factor model was still poor (CFI/TLI = 0.285/0.116, RMSEA = 0.136, SRMR = 0.270). The initial 2-factor model fit was poor with CFI/TLI: 0.478/0.414, RMSEA: 0.101, and SRMR: 0.117. Similar steps were applied and the final model turned out to be moderate fit (CFI/TLI = 0.873/0.829, RMSEA = 0.075, SRMR = 0.064). In this final 2-factor model, we added correlated items of #14 and #4, and #18 and #4. It can be seen in the table that the overall model fit for 2-factor model was better than the 5-factor or 3-factor models. 

In our final model, we had aggregated the scores for each factor and the initial model showed poor model fit (CFI/TLI = 0.634/0.267, RMSEA = 0.197, SRMR = 0.132). However, after the addition of factors with correlated uniqueness (i.e., neuroticism and conscientiousness, neuroticism and extraversion), the model fit improved substantially (CFI/TLI = 0.949/0.831, RMSEA = 0.120, SRMR = 0.038). In the final model, no items were removed and the items in each factor remained as they were. 

### 3.2. Reliability Analysis

The reliability analysis calculated for the 20 items in Mini-IPIP was 0.56 which indicates moderate or acceptable reliability [26,27].

### 3.3. Concurrent Validity

Concurrent validation for Mini-IPIP was done using ZKPQ as the gold standard (see Table 3). There were significant correlations between factors in the Mini-IPIP and the ZKPQ to at least *p*-value < 0.01 (unless indicated) except Extraversion with Impulsive-Sensation-seeking, Agreeableness with Activity, Agreeableness with Impulsive-Sensation-seeking, Agreeableness with Aggressiveness-Hostility and Agreeableness with Neuroticism-Anxiety where there was no significant association between them. 

## 4. Discussion

There is an increasing need to use a shorter form of the Five Factor Model of personality measure to limit many physical constraints associated with using longer personality measures, such as respondents’ fatigability and frustration, time constraints and error in answering the questions, all of which can lead to measurement error [5,6]. The purpose of this research was to validate a short personality questionnaire commonly used for those researchers who are willing to tolerate lower level of validity and reliability compared to their parent measures. The validation of Mini-IPIP in this study is timely in view that short personality questionnaires are needed in Malaysia for personality assessment in the busy clinical setting for patient groups with problematic cases such as substance abuse so that more comprehensive and effective interventions can be planned and executed for them. 

Our initial 5-factor CFA model of Mini-IPIP showed very poor model fit which could be due to “item cross-loadings, item residual correlations or minor factors” [7]. We did some alterations to the model with the hope to improve the model fit as “creative model re-specification” was needed in previous studies who had attained poor CFA model fit for the Big Five confirmatory analysis [9]. The model fit improved significantly, albeit remaining poor after items with poor loading were removed and items with correlated uniqueness were added into the model. The same strategies were applied to the 2- and 3-factor models as well but their model fits were not good either. Our final effort involved retaining all the items in each factor in view that each item in the Mini-IPIP was carefully selected from its parent measure to minimise inter-factorial correlations and cross-loadings to give a sharper factor structure [9]. Removing any of them would further compromise the measure as it would be difficult to cover for the facets in the personality factor. Concurrent application of the strategies of item parcelling and correlated uniqueness to our final model had resulted in a good and stable model fit. Several studies which analysed the factor structure of the 50-item IPIP-FFM using CFA had shown poor model fit initially but its model fit became good after the strategy of item parcelling was applied [28,29]. On the downside, item parcelling limits our interpretation for each item in the structure we study [8]. From the practical viewpoint, however, item parcelling allows the items in a factor to work as a group rather than as separate entities when measuring a personality dimension is concerned. This study also pointed out that for Mini-IPIP, cross-loadings do occur looking at the improvement of factor structure upon applying the strategy of correlated uniqueness. 

Using CFA to measure a model fit is sometimes argued to be too restrictive due to the potential occurrence of cross-loadings [7]. The developers for Mini-IPIP [2] also believed that it is unlikely to get a reasonable fit with CFA model for most Big Five inventories due to the strong relationship between the items in at least two factors. Cooper et al. [8] in their studies noted that their Mini-IPIP’s model fit improved after freeing items in several factors. Due to the stringent criteria applied when analysing a CFA model fit, many personality inventories failed to obtain a good model fit. Perhaps less emphasis should be placed on CFA in determining whether a model is fit or otherwise and less strict criteria should be used in CFA model fit. This may then allow the retention of items of good content without the need to remove them for the purpose of achieving the anticipated good model fit [30]. 

We believe that the limitations in our current study can offset the potential improvement for Mini-IPIP. Our suggestions to further improve on this study could perhaps enhance the results of similar studies like this in the future. Firstly, we suggest that a larger sample size be collected for a validation study. Even though the sample size for this study is deemed to be sufficient according to Hair et al. [22], there is no maximum value to the number of participants because larger sample size increases the power of study. Secondly, we suggest to use test-retest reliability, convergent and discriminant validity when analysing psychometric properties for short measures [31,32]. Finally, we suggest using a more heterogeneous representative in the population and not merely focusing on a particular subset of population. Although the male gender predominates in this study, the stability of personality measures is unlikely to be affected by sociodemographic factors such as age and gender [33,34,35].

## 5. Conclusions

Our study supported the five factor solution for Mini-IPIP when the items in each factor are taken as a whole rather than individually. This proves the complexity of human personality. Therefore, Mini-IPIP is a valid and reliable instrument to be used among individuals with substance abuse in the Malaysian setting. To have a better assessment of different facets of personality dimensions, a more extensive measure such as NEO-PI-R should be used instead. 

## Figures and Tables

**Table 1:** Mean, standard deviation and frequency (%) for sociodemographic data.

Sociodemographic Particulars	Mean (SD)	Frequency (%)
Age		
Male	39.0 (0.61)	
Female	35.8 (2.70)	
Salary	1020.9 (662.15)	
Gender		
Male		229 (95.8)
Female		10 (4.2)
Marital status		
Single		115 (48.1)
Married		92 (38.5)
Separated/divorced		32 (13.4)
Race		
Malay		219 (91.6)
Chinese		5 (2.1)
Indian		15 (6.3)
Others		0 (0)
Occupational status		
Full time		147 (61.5)
Part-time		63 (26.4)
Retired		5 (2.1)
Never worked/unemployed/Housewife		24 (10.0)
Educational level		
Never been to school		0 (0)
Primary level		11 (4.6)
Secondary level		204 (85.4)
Tertiary level		15 (6.3)
Others		9 (3.8)

**Table 2:** Confirmatory factor analysis (CFA) fit indices of various models tested for Mini-IPIP.

		Fit Indices	
Models Tested	CFI/TLI	RMSEA (90% CI)	SRMR
(a) 5 Factor			
Initial model	0.013/−0.137	0.141 (0.133–0.150)	0.232
Last model	0.600/0.472	0.102 (0.088–0.116)	0.132
(b) 3-Factor			
Initial model	0.358/0.283	0.112 (0.104–0.121)	0.253
Last model	0.285/0.116	0.136 (0.125–0.148)	0.27
(c) 2-Factor			
Initial model	0.478/0.414	0.101 (0.093–0.111)	0.117
Last model	0.873/0.829	0.075 (0.055–0.094)	0.064
(d) Aggregate score of 5 factors			
Initial model	0.634/0.267	0.197 (0.150–0.248)	0.074
Last model	0.949/0.831	0.094 (0.030–0.166)	0.044

**Table 3:** Correlations between Mini-IPIP and ZKPQ scales.

Mini-IPIP	ZKPQ				
	Activity	Impulsive-Sensation-Seeking	Sociability	Aggression-Hostility	Neuroticism-Anxiety
Intellect/Imagination	0.175 **	−0.201 **	0.283 **	−0.236 **	−0.314 **
Conscientiousness	0.295 **	−0.308 **	0.428 **	−0.412 **	−0.391 **
Extraversion	0.290 **	−0.075	0.423 **	−0.147 **	−0.209 **
Agreeableness	0.125	−0.054	0.210 **	−0.118	−0.085
Neuroticism	−0.150 *	0.174 **	−0.292 **	0.215 **	0.332 **

* *p* < 0.05, ** *p* < 0.01.
